# Development and Validation of a Prognostic Model to Predict Mortality in Patients With Heart Failure With Mildly Reduced Ejection Fraction After Acute Myocardial Infarction

**DOI:** 10.14740/cr2096

**Published:** 2026-02-28

**Authors:** Zhi Can Liu, Ling Ling Zhang, Li Peng, Jian Ping Zeng, Ming Yan Jiang

**Affiliations:** a Department of Pulmonary and Critical Care Medicine, Xiangtan Central Hospital, The Affiliated Hospital of Hunan University, Xiangtan 411100, China; b Medical Department, Xiangtan Central Hospital, The Affiliated Hospital of Hunan University, Xiangtan 411100, China; c Department of Oncology, Xiangtan Central Hospital, The Affiliated Hospital of Hunan University, Xiangtan 411100, China; d Department of Cardiology, Xiangtan Central Hospital, The Affiliated Hospital of Hunan University, Xiangtan 411100, China; e These authors contributed equally to this work.

**Keywords:** Acute myocardial infarction, Heart failure with mildly reduced ejection fraction, All-cause mortality risk, Predictive model, Least absolute shrinkage and selection operator, Decision curve analysis

## Abstract

**Background:**

Accurately assessing mortality risk in patients with heart failure with mildly reduced ejection fraction (HFmrEF) after acute myocardial infarction (AMI) remains challenging. This study developed and validated a mortality risk predictive model for such patients.

**Methods:**

In this single-center retrospective study of 873 hospitalized patients with HFmrEF after AMI, 611 patients were included in the training cohort and 262 in the validation cohort. The primary outcome was all-cause mortality over an average 33-month follow-up. Least absolute shrinkage and selection operator (LASSO) regression identified predictive variables for post-discharge mortality, with model performance assessed via receiver operating characteristic (ROC) analysis and decision curve analysis (DCA).

**Results:**

Six mortality risk predictors were identified: age, stroke history, New York Heart Association (NYHA) classification, hemoglobin (Hb) levels, estimated glomerular filtration rate (eGFR), and primary percutaneous coronary intervention (PPCI) implementation. The C-index for training and validation cohorts was 0.795 (95% confidence interval (CI), 0.758–0.832) and 0.741 (95% CI, 0.672–0.81), respectively. Training cohort area under the curve (AUC) metrics for 6-month, 2-year, and 3-year survival were 0.861, 0.805, and 0.815; for the validation cohort, they were 0.722, 0.742, and 0.736.

**Conclusions:**

A validated predictive model assessing mortality risk in HFmrEF patients post-AMI was established. External validation in future studies is recommended.

## Introduction

Cardiovascular diseases, especially heart failure (HF), impose a substantial burden on global health [1]. Following technical and medication advances, acute mortality of acute myocardial infarction (AMI) declined substantially, but patients who survived AMI often faced increased risk of HF [2–5]. Among HF patients, those with a mildly reduced ejection fraction (HFmrEF) warrant focused attention due to their non-benign prognosis and the potential progression to HF with reduced ejection fraction [6, 7]. Intriguingly, the prognosis may deviate for HFmrEF patients following AMI compared to HFmrEF patients from other etiologies [8–11]. Predicting mortality risk for HFmrEF patients post AMI is thus of clinical importance.

There are various prognostic models for the prediction of survival rates in HF patients [12, 13]; nevertheless, predictive models of mortality for HFmrEF patients subsequent to AMI are still lacking.

In response to these gaps, we aimed to construct and validate a predictive model to assess the mortality risk in HFmrEF patients post AMI. The primary endpoint was all-cause mortality during follow-up.

## Materials and Methods

### Study design

**Figure 1:**
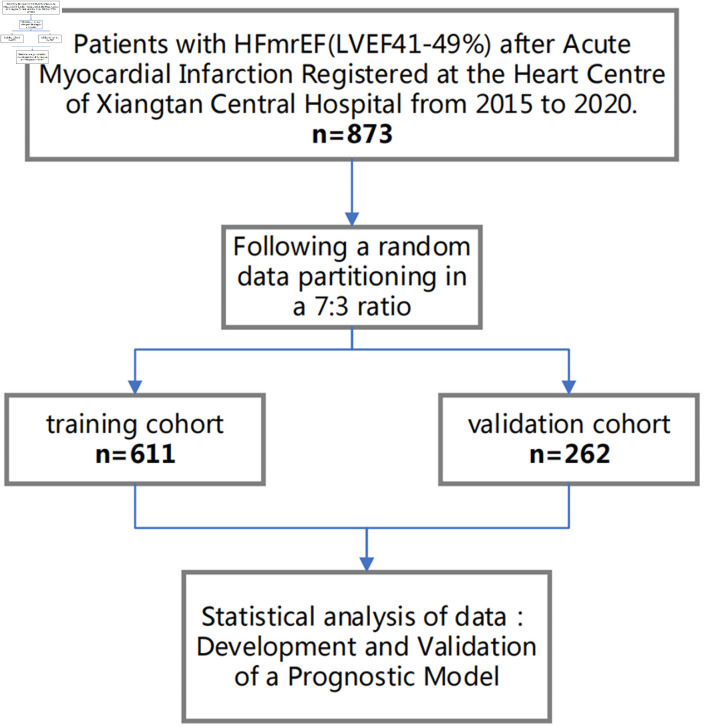
Flow diagram for participant screening, eligibility, and analysis. HFmrEF: heart failure with mildly reduced ejection fraction; LVEF: left ventricular ejection fraction.

Hospitalized HFmrEF patients with a documented history of AMI between January 1, 2015, and August 31, 2020, in our hospital were eligible for this single-center retrospective study. The inclusion criteria were a documented history of AMI, a left ventricular ejection fraction (LVEF) ranging from 41% to 49% as measured by transthoracic echocardiography performed during the index hospitalization (post-AMI and prior to discharge), and New York Heart Association (NYHA) functional classes II to IV. Patients diagnosed with malignant neoplasms or any non-cardiac related disease that projected a life expectancy of less than 1 year were excluded. After meticulous screening, 873 patients were included, and patients were randomly divided into a training set (comprising 611 patients) and a validation set (comprising 262 patients) at a 7:3 ratio (Fig. 1). Ethical approval for this study was obtained from the Ethics Committee of Xiangtan Central Hospital (Approval No. 20211036), and the study was conducted in accordance with the principles of the Declaration of Helsinki. Given the retrospective nature of this study, which solely entailed the aggregation of clinical data without influencing patient treatment trajectories, the requirement for informed consent was waived.

### Data collection

Baseline characteristics of patients, including age, sex, medical history, laboratory examination outcomes, treatment modalities, and so forth, were extracted from the hospital’s integrated electronic health record system. The primary outcome measure, focusing on the all-cause mortality after hospital discharge, was primarily retrieved from detailed medical archives, clinical visits, and community visits or telephone interviews.

### Primary outcome

The principal outcome of interest was the post-discharge all-cause mortality rate. Follow-up commenced immediately upon hospital discharge. Data about mortality were compiled from the hospital’s electronic health record system, and patient follow-ups ended until August 31, 2021. The median follow-up duration was 33 months. Ascertainment of the outcome event involved a comprehensive examination of hospital records and subsequent interventions, including but not limited to clinical consultations, telephonic interviews, and community outreach programs. The follow-up team comprised a panel of seven healthcare professionals, including physicians and nurses.

### Statistical analysis

All datasets underwent normalization via z-score transformation, which yielded a mean value of 0 and a standard deviation of 1. The least absolute shrinkage and selection operator (LASSO) regression methodology was employed to discern variables that exhibited a significant correlation with all-cause mortality. A regression model encapsulating these identified variables was developed using the “glmnet” package in R language, which is specifically designed for LASSO regression modeling. Each patient’s mortality risk score was computed through a linear combination of the selected predictive variables and their respective coefficients. The optimal lambda (λ) parameter, which minimizes the cross-validation error, was selected. Efforts were made to reduce covariate coefficients to 0 during the refitting of the model using the chosen λ and all available observations. Non-zero coefficients identified by the LASSO procedure were retained and categorized as mortality risk predictors. Subsequently, mortality risk prediction nomograms were developed using the “rms” package of R language.

The model’s performance was evaluated through discrimination and calibration analyses. Discrimination was quantified by the area under the receiver operating characteristic (ROC) curve, while calibration was assessed via calibration plots. The net benefit of the model was determined through decision curve analysis (DCA). The predictive accuracy of the risk model was evaluated using the C-statistic for discrimination, and calibration was assessed using calibration plots and Brier scores.

Depending on the context, intergroup differences were evaluated using independent Student’s *t*-tests, Chi-square tests, or Mann–Whitney U tests. The Kolmogorov–Smirnov test was employed to ascertain normality. Continuous variables adhering to a normal distribution were reported as mean values ± standard deviation, while non-normally distributed continuous variables were expressed as median values with interquartile ranges. Categorical variables were presented as n (%). All statistical tests were two-tailed, and a P value less than 0.05 was considered statistically significant. Similar methods were used to assess the performance in terms of model development, discrimination, and calibration. All statistical analyses were performed using R software, version 4.2.0.

## Results

Demographically, patients in the training and validation groups exhibited a median age of 69.0 years. There were 71.0% male patients in the training cohort and 66.4% in the validation cohort. The median body mass index (BMI) for both cohorts was identical (24.4 kg/m^2^). The prevalence of patients with NYHA functional classification III + IV was 53.0% and 46.9% in the training and validation cohorts, respectively (all P > 0.05) (Table 1).

**Table 1:** Baseline Characteristics of Patients With HFmrEF After Acute Myocardial Infarction in the Mortality Prognostic Model Development and Validation

	Training cohort (n = 611)	Validation cohort (n = 262)	P value
Demographics			
Age, years	69.0 (61.0; 77.0)	69.0 (61.0; 77.0)	0.734
Male, n (%)	434 (71.0%)	174 (66.4%)	0.201
Body mass index, kg/m^2^	24.4 (22.8; 30.0)	24.4 (22.9; 30.0)	0.988
NYHA III + IV, n (%)	324 (53.0%)	123 (46.9%)	0.116
Medical history, n (%)			
Current smoker	221 (36.2%)	95 (36.3%)	1.000
Current drinker	53 (8.67%)	26 (9.92%)	0.645
Hypertension	432 (70.7%)	168 (64.1%)	0.065
Hyperlipidemia	126 (20.6%)	57 (21.8%)	0.775
Coronary heart disease	605 (99.0%)	261 (99.6%)	0.681
Atrial fibrillation	71 (11.6%)	28 (10.7%)	0.778
Stroke	77 (12.6%)	29 (11.1%)	0.601
COPD	66 (10.8%)	22 (8.40%)	0.338
Renal insufficiency	127 (20.8%)	42 (16.0%)	0.124
Diabetes	206 (33.7%)	96 (36.6%)	0.450
Serology			
NT-proBNP/100, pg/mL	17.7 (4.50; 57.5)	21.1 (4.36; 63.3)	0.797
Hemoglobin, g/L	126 (112; 139)	130 (114; 142)	0.164
White blood cells, × 10^9^/L	8.33 (6.35; 10.8)	8.41 (6.42; 10.7)	0.959
Platelets, × 10^9^/L	187 (150; 227)	187 (148; 231)	0.908
Alanine aminotransferase, U/L	25.7 (14.9; 43.8)	26.9 (14.5; 46.9)	0.756
Aspartate aminotransferase, U/L	32.1 (20.1; 150)	36.2 (21.1; 134)	0.413
Creatine kinase-MB, U/L	24.5 (14.0; 88.2)	25.9 (14.8; 113)	0.550
Uric acid, µmol/L	340 (276; 416)	332 (264; 415)	0.378
eGFR, mL/min/1.73 m^2^	73.9 (51.0; 93.7)	73.7 (57.6; 93.2)	0.680
Sodium, mg/L	140 (137; 142)	140 (137; 141)	0.972
Potassium, mg/L	4.12 (3.80; 4.48)	4.08 (3.77; 4.40)	0.248
Low-density lipoprotein, mmol/L	2.42 (1.73; 3.13)	2.50 (1.78; 3.18)	0.280
Treatment, n (%)			
PPCI	392 (64.2%)	173 (66.0%)	0.122
Beta-blocker	534 (87.4%)	235 (89.7%)	0.397
ACEI or ARB	498 (81.5%)	221 (84.4%)	0.361
ARNI	34 (5.56%)	12 (4.58%)	0.666
SGLT2i	2 (0.33%)	1 (0.38%)	1.000
Diuretics	319 (52.2%)	130 (49.6%)	0.530
Spironolactone	292 (47.8%)	124 (47.3%)	0.959
Echocardiography			
Aortic size, mm	33.0 (31.0; 36.0)	33.5 (31.0; 36.0)	0.762
Left atrium size, mm	37.0 (34.0; 40.0)	37.0 (35.0; 40.0)	0.992
LVd, mm	51.0 (48.0; 55.0)	51.0 (47.0; 55.0)	0.160
Right atrium size, mm	35.0 (33.0; 38.0)	36.0 (34.0; 38.0)	0.023
RVd, mm	19.0 (17.0; 20.0)	19.0 (18.0; 20.0)	0.176
E/e’, cm/s	13.3 (9.80; 18.4)	13.7 (10.4; 18.0)	0.396
PASP, mm Hg	31.0 (18.0; 40.0)	31.0 (22.0; 39.8)	0.884
Clinical endpoints, n (%)			
Death	128 (20.9%)	55 (21.0%)	1.000

According to a 7:3 ratio, the population was randomly divided into a training cohort and a validation cohort. Categorical variables were presented as n (%). Values for continuous variables are given as means ± SD or medians with interquartile ranges. HFmrEF: heart failure with mildly reduced ejection fraction; NYHA: New York Heart Association; COPD: chronic obstructive pulmonary disease; ACEI: angiotensin-converting enzyme inhibitor; ARB: angiotensin receptor blocker; ARNI: angiotensin receptor-neprilysin inhibitor; SGLT2i: sodium–glucose cotransporter 2 inhibitors; NT-proBNP: N-terminal pro-brain natriuretic peptide; eGFR: estimated glomerular filtration rate; LVd: left ventricular end-diastolic diameter; RVd: right ventricular end-diastolic diameter; E/e’: early diastolic peak velocity/early diastolic peak velocity of the lateral or septal mitral annulus; PASP: pulmonary artery systolic pressure; SD: standard deviation.

Baseline features including percent of current smokers, excessive alcohol consumers, and diagnoses of hypertension, hyperlipidemia, coronary artery disease, atrial fibrillation, stroke, chronic obstructive pulmonary disease (COPD), renal insufficiency, and diabetes were similar between the two cohorts (all P values > 0.05). Similarly, serum biomarkers such as N-terminal pro-brain natriuretic peptide (NT-proBNP)/100, hemoglobin, white blood cells, platelets, alanine aminotransferase, aspartate aminotransferase, creatine kinase-MB, uric acid, estimated glomerular filtration rate (eGFR), sodium, potassium, and low-density lipoprotein were also similar between the two cohorts (all P values > 0.05). Likewise, interventions and prescriptions—including primary percutaneous coronary intervention (PPCI), β-blockers, angiotensin-converting enzyme inhibitors (ACEIs) or angiotensin receptor blockers (ARBs), angiotensin receptor-neprilysin inhibitors (ARNIs), sodium–glucose cotransporter 2 (SGLT2) inhibitors, diuretics, and spironolactone—were comparably distributed between the groups (all P values > 0.05). Echocardiography derived parameters, including aortic dimensions, left atrial size, left ventricular end-diastolic diameter (LVd), right atrial dimensions, right ventricular end-diastolic diameter (RVd), E/e’ ratio, and pulmonary artery systolic pressure (PASP), were consistent across the two cohorts. However, a significant discrepancy was observed regarding the right atrial dimension, with a P value of 0.023. Clinical endpoints revealed mortality rates of 20.9% and 21.0% within the training and validation cohorts, respectively (P = 1.000) (Table 1).

Univariate Cox regression showed that age (hazard ratio (HR) = 1.08; 95% confidence interval (CI), 1.061–1.1; P < 0.001), diabetes (HR = 1.476; 95% CI, 1.037–2.1; P = 0.031), renal dysfunction (HR = 2.857; 95% CI, 1.995–4.09; P < 0.001), COPD (HR = 2.236; 95% CI, 1.443–3.463; P < 0.001), stroke (HR = 2.808; 95% CI, 1.888–4.175; P < 0.001), atrial fibrillation (HR = 1.982; 95% CI, 1.27–3.092; P = 0.003), hypertension (HR = 2.187; 95% CI, 1.381–3.462; P = 0.001), and NYHA class III + IV (as opposed to NYHA class II, HR = 2.735; 95% CI, 1.84–4.065; P < 0.001), in conjunction with specific laboratory (e.g., uric acid, NT-proBNP/100, PASP, E/e’) and echocardiographic markers (such as LVd, left atrial dimension, and aortic dimension), were predictors for increased risk of mortality (Table 2).

**Table 2:** Univariate Cox Regression Analysis of Factors Associated With Mortality in Patients With HFmrEF After Acute Myocardial Infarction

characteristics	β	SE	HR	95% CI	Z score	P value
Age	0.077	0.009	1.08	1.061–1.1	8.297	< 0.001^a^
Male vs. female	0.074	0.198	1.076	0.73–1.588	0.371	0.711
Body mass index	–0.004	0.022	0.996	0.954–1.041	–0.159	0.874
Current smoker	–0.263	0.191	0.769	0.529–1.117	–1.378	0.168
Current drinker	–0.234	0.329	0.791	0.415–1.51	–0.71	0.478
Diabetes	0.389	0.18	1.476	1.037–2.1	2.16	0.031^a^
Renal insufficiency	1.05	0.183	2.857	1.995–4.09	5.731	< 0.001^a^
COPD	0.804	0.223	2.236	1.443–3.463	3.603	< 0.001^a^
Stroke	1.032	0.202	2.808	1.888–4.175	5.1	< 0.001^a^
Atrial fibrillation	0.684	0.227	1.982	1.27–3.092	3.015	0.003^a^
Hyperlipidemia	–0.617	0.261	0.539	0.324–0.899	–2.369	0.018^a^
Hypertension	0.782	0.234	2.187	1.381–3.462	3.338	0.001^a^
NYHA III + IV vs. NYHA II	1.006	0.202	2.735	1.84–4.065	4.974	< 0.001^a^
Low-density lipoprotein	–0.153	0.095	0.858	0.713–1.034	–1.608	0.108
Sodium	0.05	0.152	1.052	0.781–1.416	0.332	0.74
Potassium	0.013	0.025	1.013	0.966–1.063	0.533	0.594
eGFR	–0.026	0.003	0.974	0.968–0.98	–8.33	< 0.001^a^
Uric acid	0.002	0.001	1.002	1.001–1.004	3.277	0.001^a^
Creatine kinase-MB	–0.003	0.001	0.997	0.995–0.999	–3.038	0.002^a^
Alanine aminotransferase	–0.002	0.001	0.998	0.996–0.999	–2.716	0.007^a^
Aspartate aminotransferase	–0.011	0.004	0.99	0.982–0.997	–2.642	0.008^a^
White blood cells	–0.056	0.027	0.946	0.897–0.997	–2.063	0.039^a^
Hemoglobin	–0.032	0.004	0.969	0.962–0.976	–8.512	< 0.001^a^
NT-proBNP/100	0.005	0.001	1.005	1.003–1.006	5.919	< 0.001^a^
PASP	0.01	0.002	1.01	1.007–1.013	5.826	< 0.001^a^
E/e’	0.052	0.011	1.053	1.03–1.077	4.562	< 0.001^a^
RVd	0.028	0.022	1.028	0.985–1.073	1.282	0.2
Right atrium size	0.029	0.017	1.029	0.996–1.063	1.739	0.082
LVd	0.041	0.014	1.042	1.013–1.072	2.887	0.004^a^
Left atrium size	0.076	0.015	1.079	1.048–1.111	5.121	< 0.001^a^
Aortic size	0.047	0.024	1.048	1–1.098	1.973	0.049^a^
PPCI	–1.039	0.18	0.354	0.249–0.504	–5.772	< 0.001^a^
Diuretics	0.262	0.179	1.3	0.915–1.847	1.464	0.143
ARNI	0.343	0.423	1.409	0.616–3.226	0.812	0.417
ACEI or ARB	–1.032	0.191	0.356	0.245–0.518	–5.402	< 0.001^a^
Spironolactone	–0.141	0.178	0.868	0.613–1.229	–0.797	0.426
Beta-blocker	–0.913	0.209	0.401	0.266–0.604 –	–4.37	< 0.001^a^

^a^P < 0.05. HFmrEF: heart failure with mildly reduced ejection fraction; NYHA: New York Heart Association; COPD: chronic obstructive pulmonary disease; ACEI: angiotensin-converting enzyme inhibitors; ARB: angiotensin receptor blocker; ARNI: angiotensin receptor-neprilysin inhibitor; SGLT2i: sodium–glucose cotransporter 2 inhibitors; PPCI: primary percutaneous coronary intervention; NT-proBNP: N-terminal pro-brain natriuretic peptide; eGFR: estimated glomerular filtration rate; LVd: left ventricular end-diastolic diameter; RVd: right ventricular end-diastolic diameter; E/e’: early diastolic peak velocity/early diastolic peak velocity of the lateral or septal mitral annulus; PASP: pulmonary artery systolic pressure; SE: standard error; HR: hazard ratio; CI: confidence interval.

Conversely, parameters including high-density lipoprotein, eGFR, creatine kinase-MB, alanine aminotransferase, aspartate aminotransferase, white blood cell count, hemoglobin, PPCI, ACEIs or ARBs, and β-blockers were inversely associated with increased risk of mortality. Notably, factors such as sex, BMI, current smoking and drinking status, hyperlipidemia, serum sodium and potassium levels, RVd, right atrial dimensions, and diuretic, ARNIs, and spironolactone administration were not linked with mortality outcomes (Table 2).

**Figure 2:**
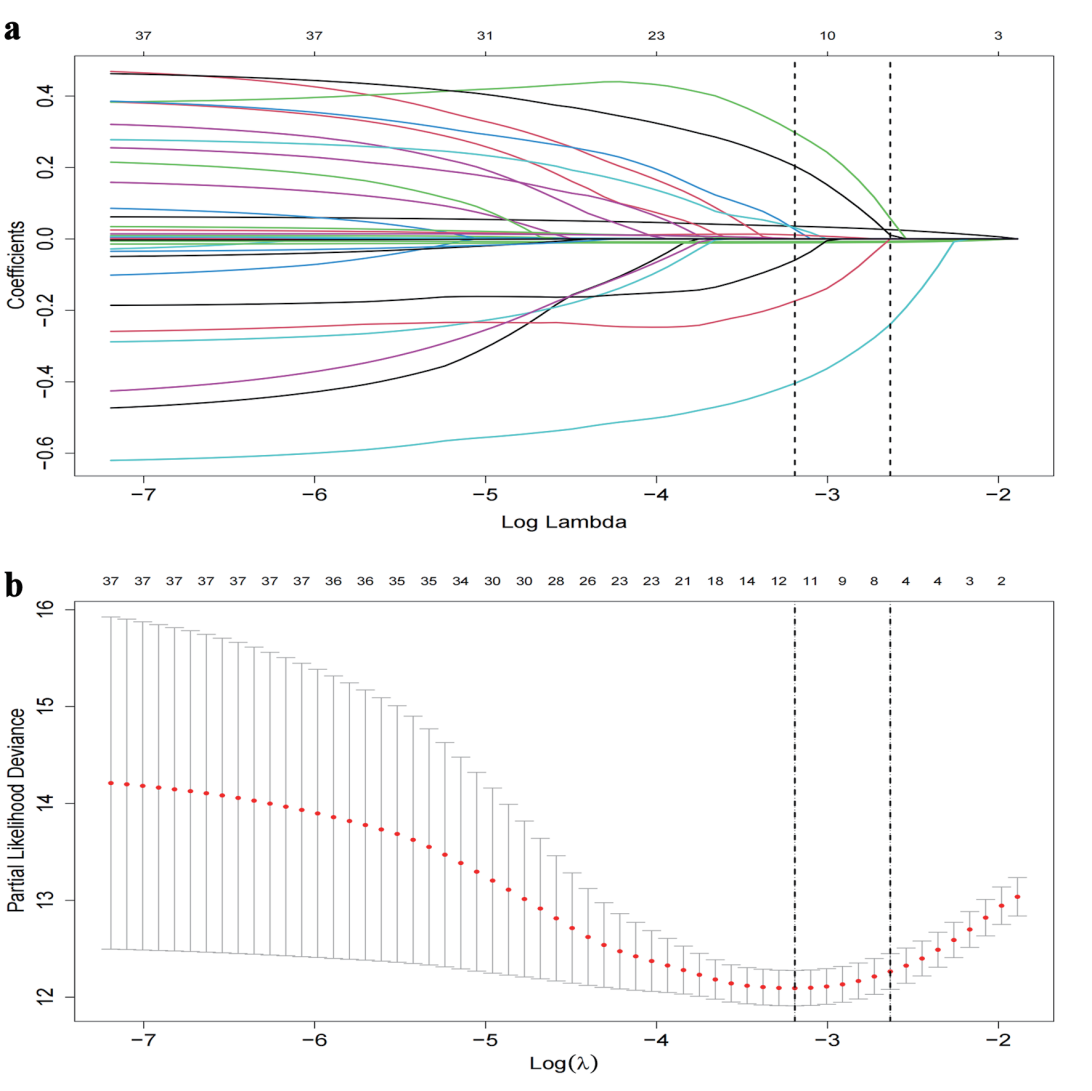
LASSO regression coefficient path and CV LASSO regression coefficient path. (a) LASSO regression coefficient path. (b) CV LASSO regression coefficient path. The LASSO regression coefficient path displays how the coefficients of each variable change with increasing values of the regularization parameter λ. The CV LASSO regression coefficient path illustrates the coefficients’ behavior with λ tuned through cross-validation. Both paths provide insights into the impact of regularization on variable selection and coefficient estimation in the LASSO regression model. LASSO: least absolute shrinkage and selection operator.

Utilizing the LASSO regression methodology, we identified six pivotal risk factors of mortality: age, hemoglobin levels, eGFR, PPCI, prior stroke events, and NYHA functional classification of III + IV (Fig. 2).

Results from the Cox proportional-hazards regression analysis, based on the six variables derived from the LASSO regression, are presented as follows (Table 3): age (HR = 1.053; 95% CI, 1.034–1.073; P < 0.001), history of stroke (HR = 1.69; 95% CI, 1.13–2.528; P = 0.011), and NYHA class III + IV (relative to NYHA class II, HR = 1.673; 95% CI, 1.111–2.519; P = 0.014) showed substantial associations with an increased mortality risk. Conversely, factors such as hemoglobin concentration (HR = 0.984; 95% CI, 0.975–0.993; P = 0.001), eGFR (HR = 0.988; 95% CI, 0.981–0.996; P = 0.003), and PPCI (HR = 0.552; 95% CI, 0.383–0.797; P = 0.001) were correlated with a reduced risk of mortality.

**Table 3:** Multivariable Cox Regression Analysis of Factors Associated With Mortality in HFmrEF Patients

Characteristics	β	SE	HR	95% CI	Z score	P value
Age	0.052	0.009	1.053	1.034–1.073	5.555	< 0.001^a^
Hemoglobin	–0.016	0.005	0.984	0.975–0.993	–3.42	0.001^a^
eGFR	–0.012	0.004	0.988	0.981–0.996	–2.98	0.003^a^
PPCI	–0.594	0.187	0.552	0.383–0.797	–3.176	0.001^a^
Stroke	0.525	0.205	1.69	1.13–2.528	2.556	0.011^a^
NYHA III + IV vs. NYHA II	0.514	0.209	1.673	1.111–2.519	2.462	0.014^a^

^a^P < 0.05. HFmrEF: heart failure with mildly reduced ejection fraction; eGFR: estimated glomerular filtration rate; PPCI: primary percutaneous coronary intervention; NYHA: New York Heart Association; SE: standard error; HR: hazard ratio; CI: confidence interval.

**Figure 3:**
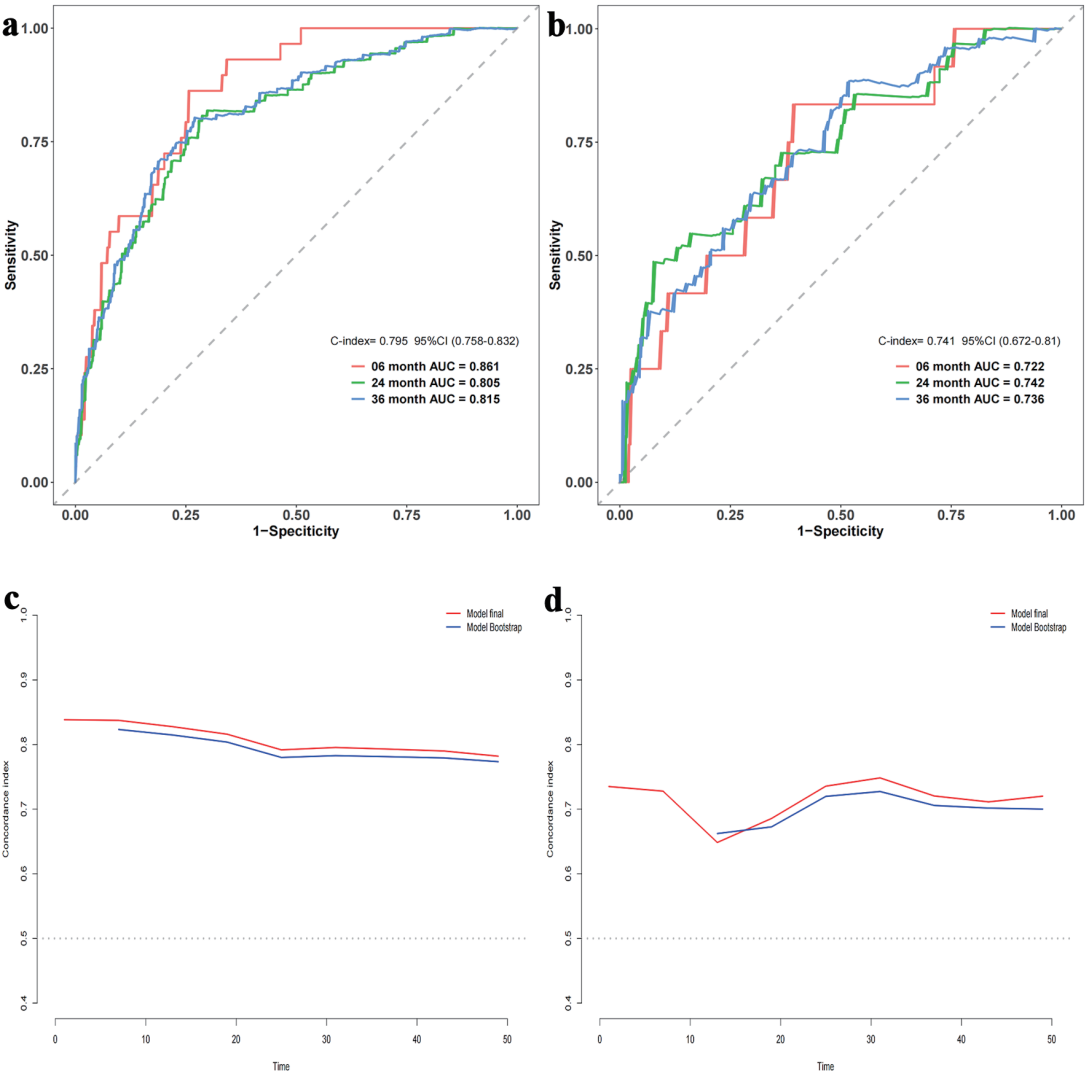
Area under the receiver operating characteristic (ROC) curve and bootstrap validation. (a) ROC curves for the training set at 6 months, 2 years, and 3 years. (b) ROC curves for the validation set at 6 months, 2 years, and 3 years. (c) Comparison of model stability between the original model and 500 rounds of bootstrap validation on the training set. (d) Comparison of model stability between the original model and 500 rounds of bootstrap validation on the validation set. AUC: area under the curve; CI: confidence interval.

**Figure 4:**
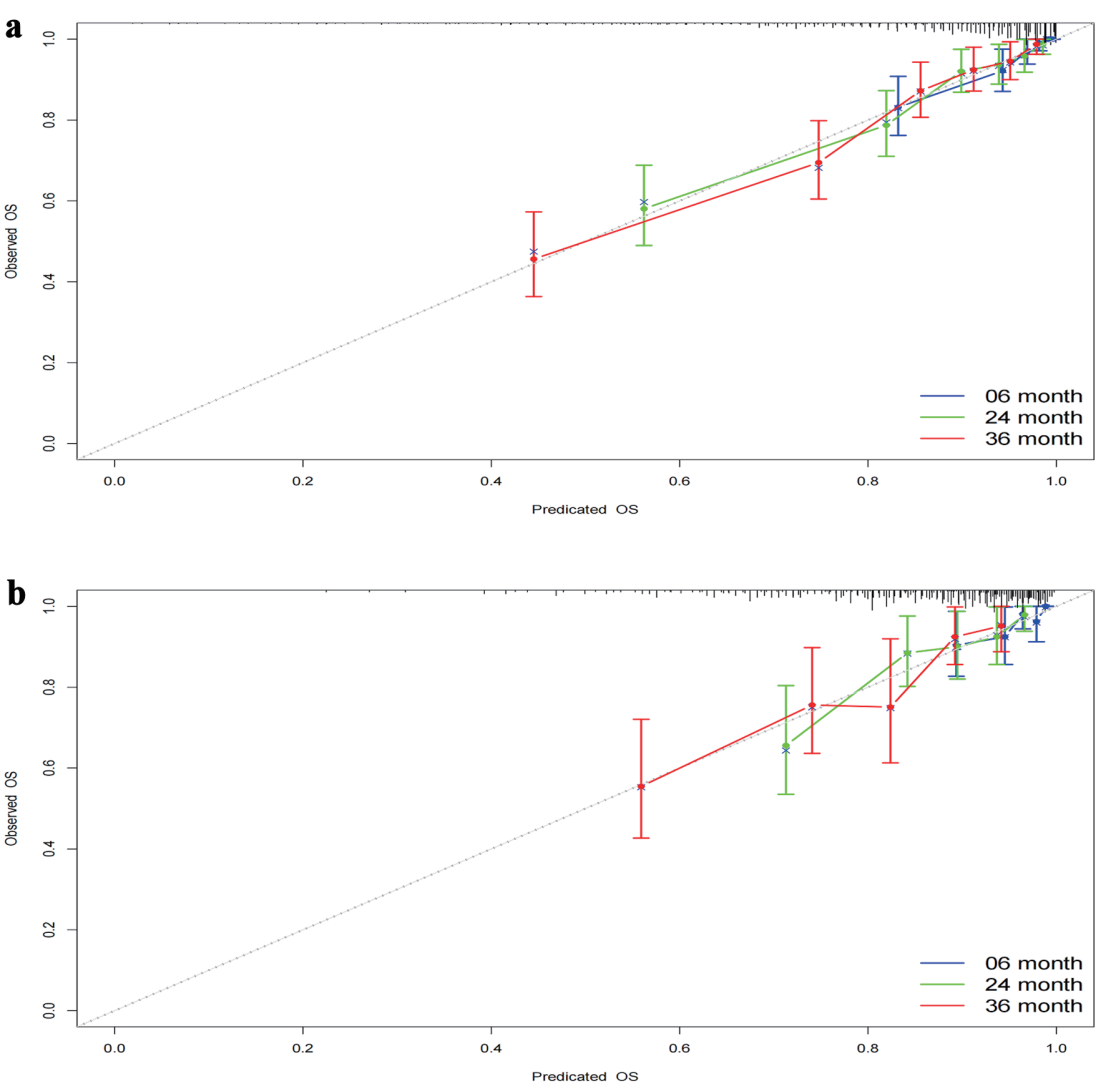
Calibration curves at different time points. (a) Calibration curves for the training set at 6 months, 2 years, and 3 years. (b) Calibration curves for the validation set at 6 months, 2 years, and 3 years. The calibration curves depict the agreement between the predicted probabilities and the observed outcomes at different time points. The curves represent the performance of the predictive model in terms of calibration, indicating how well the model’s predicted probabilities align with the actual probabilities. OS: overall survival.

Time-dependent ROC curves were constructed to elucidate the discriminatory power of our model. A C-index of 0.795 with a 95% CI of 0.758–0.832 was observed for the training cohort. The area under the curve (AUC) values were determined to be 0.861 at 6 months, 0.805 at 2 years, and 0.815 at 3 years. For the validation cohort, a C-index of 0.741 with a 95% CI of 0.672–0.81 was noted, and AUCs were 0.722, 0.742, and 0.736, respectively (Fig. 3a, c). After 500 bootstrap resampling iterations, the stability of models across both cohorts was affirmed (Fig. 3b, d). Calibration plots demonstrated excellent calibration for both cohorts at the designated time points of 6 months, 2 years, and 3 years (Fig. 4), underscoring the model’s reliability. The corresponding Brier scores were 0.073, 0.145, and 0.170, respectively, further confirming satisfactory predictive performance.

**Figure 5:**
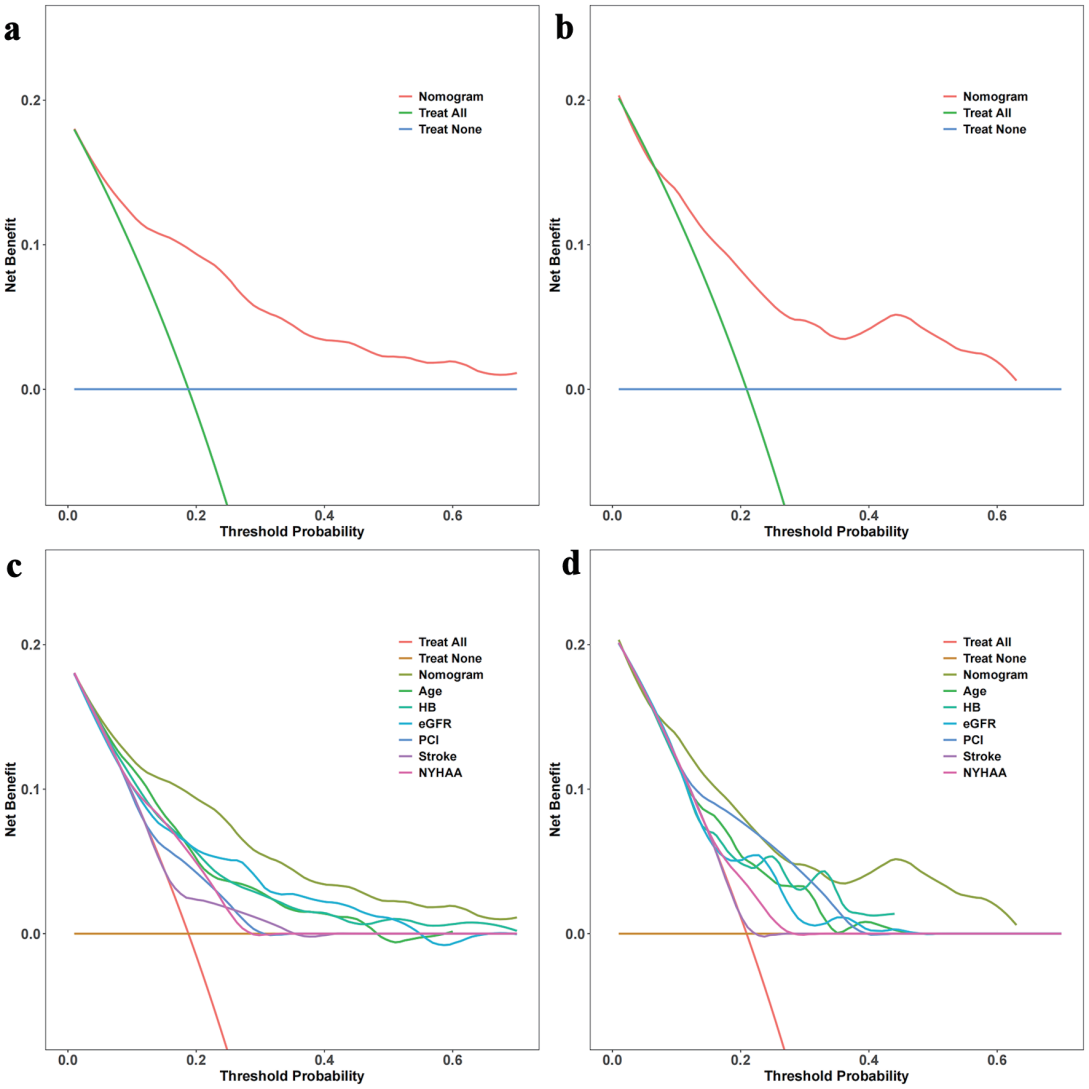
Decision curve analysis (DCA) with time and DCA nomogram. (a) DCA with time for the training set. (b) DCA with time for the validation set. (c) DCA nomogram for the training set. (d) DCA nomogram for the validation set. The DCA curves in Figure 5a and 5b illustrate the net benefit of the predictive model over a range of threshold probabilities at different time points for the training and validation sets. These curves provide insights into the clinical usefulness and added value of the model compared to alternative decision strategies. Additionally, the DCA nomograms in Figure 5c and 5d provide a graphical representation of the decision curves, allowing for a more intuitive interpretation and application of the model’s results. Hb: hemoglobin; NYHA: New York Heart Association; eGFR: estimated glomerular filtration rate; PCI: percutaneous coronary intervention.

**Figure 6:**
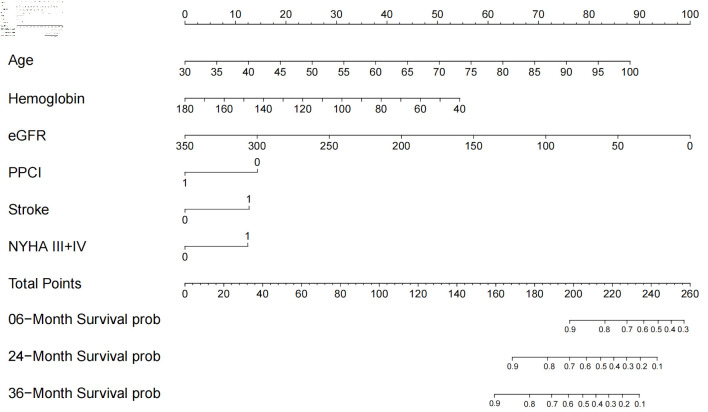
Nomogram for all-cause mortality risk prediction. The nomogram presents a visual tool for predicting the risk of all-cause mortality. It combines various predictors or risk factors into a comprehensive model that provides an individualized risk assessment. The nomogram allows for a simple and intuitive estimation of the probability of mortality based on the values assigned to each predictor. Clinicians can use this nomogram as a practical aid in risk assessment and shared decision-making with patients regarding appropriate interventions and management strategies. NYHA: New York Heart Association; eGFR: estimated glomerular filtration rate; PPCI: primary percutaneous coronary intervention.

**Figure 7:**
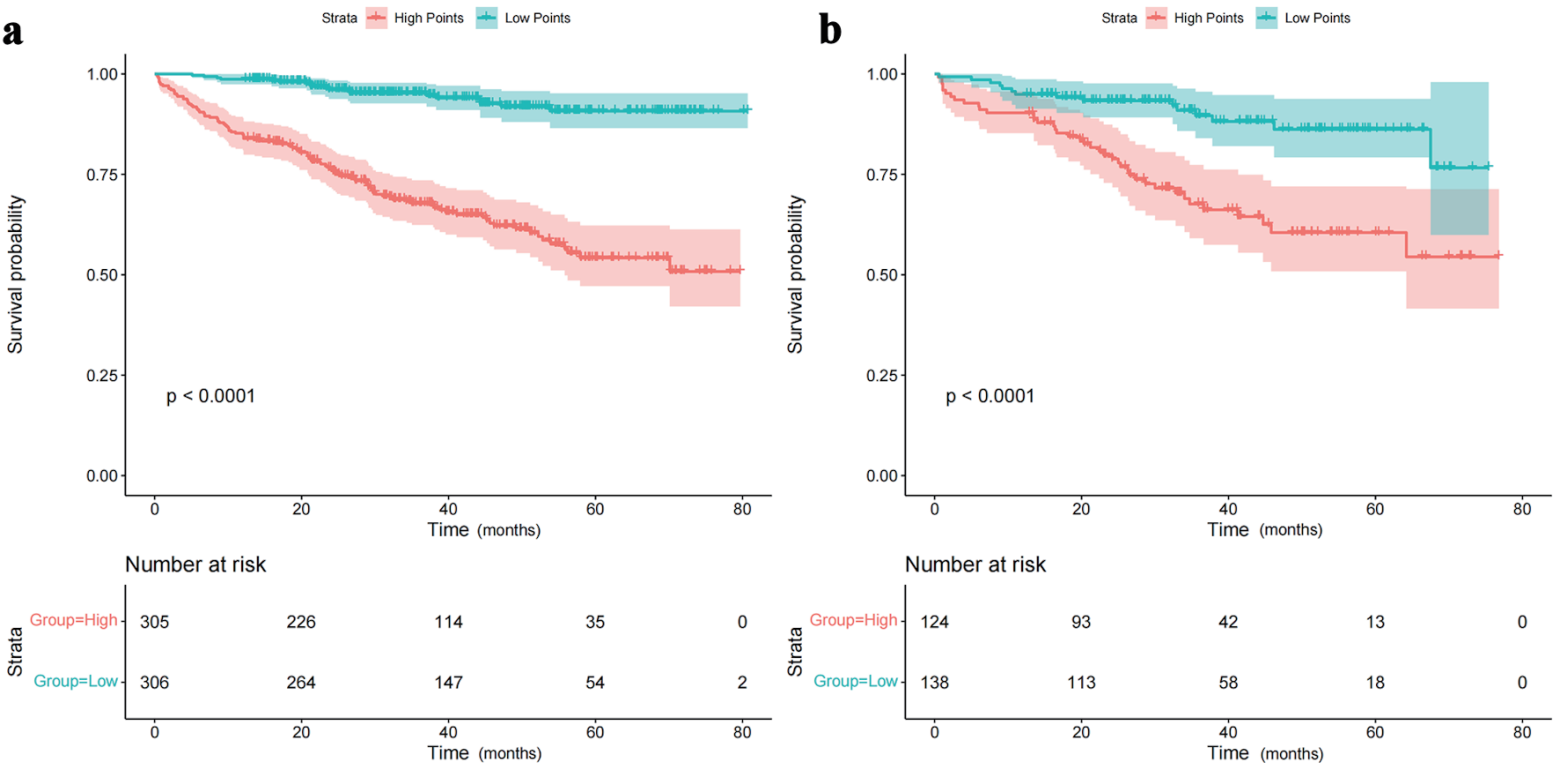
Rationality analysis: Kaplan-Meier survival curves of high-score and low-score groups. (a) Rationality analysis for the training set. (b) Rationality analysis for the validation set. The Kaplan-Meier survival curves depicted in Figure 7a and 7b demonstrate the differences in survival outcomes between the high-score and low-score groups. These curves serve as a rationality analysis to evaluate the predictive performance of the scoring system or model. The separation of the survival curves indicates the ability of the scoring system to stratify patients into distinct risk groups. This analysis provides insights into the reliability and validity of the scoring system in predicting survival outcomes and aids in assessing its clinical utility.

The time-dependent DCA and the DCA nomogram indicated a net prognostic benefit across the cohorts, with the nomogram displaying clear superiority over the individual six parameters (Fig. 5). Figure 6 showcases a line chart illustrating the risk scores corresponding to the six prognostic determinants; higher scores signify an increased prospective mortality risk. Using this chart for risk stratification, patients were categorized into high-score and low-score groups. Kaplan-Meier survival analyses confirmed that, irrespective of the training or validation cohorts, individuals in the low-score group consistently exhibited a reduced mortality risk compared to those in the high-score group (Fig. 7).

## Discussion

The principal finding of this study is the successful development of a tailored prognostic tool that quantifies mortality risk in the specific, yet often overlooked, population of patients with HFmrEF following AMI. By employing LASSO regression to ensure model parsimony, we identified six distinct predictors—age, NYHA class, hemoglobin, eGFR, PPCI, and stroke history—that effectively capture the multidimensional nature of risk in this cohort. Rather than being solely defined by cardiac metrics, our results suggest that prognosis in the post-AMI HFmrEF setting is driven by a complex interplay between acute ischemic intervention (PPCI), functional status (NYHA), and systemic comorbidities (renal dysfunction, anemia, and cerebrovascular history). This highlights that survival in these patients depends not only on cardiac recovery but also on the comprehensive management of non-cardiac burden.

Existing literature consistently identifies age as an independent risk factor for cardiovascular diseases and mortality [14], a finding that aligns with our observations. Similarly, stroke has been shown to be linked with increased incidences of HF and elevated mortality rates [15], corroborating our findings. The NYHA functional classification, a recognized indicator for charting the progression of HF, demonstrates a negative correlation with survival rates, in line with our observations [16].

Several variables, including hemoglobin level, eGFR, and PPCI, have been investigated as potential prognostic markers for HF and other cardiovascular disorders in numerous studies [17–20]. However, their specific roles in patients with HFmrEF following an AMI remain unclear. For instance, although a low eGFR is universally accepted as being associated with increased risk of HF and other cardiovascular diseases, its relationship with HFmrEF following AMI remains to be clarified. PPCI, an established procedure for revascularization, is widely used in treating acute coronary syndrome [21] and has been demonstrated to improve prognosis [22]. However, its precise role in patients with HFmrEF post-AMI remains uncertain. Our research provides novel insights into the roles of these variables in this unique patient population.

Notably, several potential predictors, such as hypertension, hyperlipidemia, and coronary artery disease, demonstrated a significant correlation with increased mortality in our univariate analysis but were excluded from the final model following LASSO regression. This could be due to their collinearity with other more significant predictors, or possibly their less predictive value within this specific patient group. It is worth noting that the utilization of SGLT2 inhibitors in our cohort was extremely low (< 1%). This reflects the historical context of the study period (2015–2020), during which SGLT2 inhibitors were not yet standard of care for non-diabetic HF patients. The pivotal evidence supporting their use in HFmrEF and subsequent guideline recommendations emerged after the conclusion of our study enrollment. Consequently, our data represent the real-world clinical practice for post-AMI HFmrEF patients prior to the “SGLT2 inhibitor era.”

Although established risk scores such as the Seattle Heart Failure Model (SHFM) and the MAGGIC risk score are widely used for HF prognosis, their applicability specifically to HFmrEF patients in the post-AMI setting remains underinvestigated [12, 13]. These general scores were largely derived from chronic HF populations or mixed cohorts where the specific phenotype of ischemic HFmrEF was underrepresented. In contrast, our model focuses specifically on the distinct profile of post-AMI HFmrEF, capturing the intersection of acute ischemic injury and pump failure. By utilizing only six readily available clinical variables, our nomogram balances predictive accuracy with clinical utility, offering a tailored tool for this specific high-risk subgroup, where general models—which may require complex variables or lack acute-phase specific factors like PPCI—might be less practical or precise.

Furthermore, while it is well established that maximal oxygen consumption (VO_2_) measured by cardiopulmonary exercise testing (CPET) is the most extensively studied marker and predictor for prognosis and risk stratification in HF [23, 24], its clinical application is often limited in patients with physical disabilities or those unable to tolerate exercise stress. Therefore, our prognostic model may be particularly useful for these vulnerable subgroups, offering a reliable risk assessment tool that does not rely on functional capacity testing.

Finally, recent evidence suggests that the CHA_2_DS_2_-VASc score, traditionally used for stroke risk stratification in atrial fibrillation, may also hold prognostic value for HF patients, even in the absence of atrial fibrillation [25, 26]. Future studies are warranted to explore whether this score provides additional predictive value specifically in HFmrEF populations, potentially complementing our proposed nomogram for more comprehensive risk stratification.

### Clinical implication of the study

Our results might be helpful for the risk stratification of HFmrEF patients following an AMI. Special care and monitoring should be applied to patients with advanced age, higher NYHA functional classification, lower hemoglobin levels, eGFR, past history of stroke and patients without PPCI. Targeted intervention should be considered to address the modifiable risk factors among these patients. Future studies are needed to estimate if correction of anemia, prevention of renewed onset of stroke, improvement of kidney function, rehabilitation intervention, consideration of PPCI could improve the outcome of these patients or not.

### Limitations of the study

Our study has several limitations that should be acknowledged. First, it is a retrospective, single-center study, which may have introduced potential selection bias. Future investigations should employ multi-center, prospective designs to validate our findings.

Second, LVEF was assessed only during the index hospitalization. Given the dynamic nature of cardiac function after AMI, some patients may have experienced LVEF recovery or deterioration during the follow-up period. Our model relies on baseline characteristics to predict outcomes and does not account for longitudinal changes in LVEF or reclassify patients based on follow-up echocardiography. Future studies using time-dependent covariates could provide further insights.

Third, treatment variables (including PPCI and medications) were analyzed as binary, static baseline variables recorded at the index hospitalization. We lacked granular data on medication dosage, long-term adherence, or treatment changes during the follow-up period. Consequently, our analysis does not account for time-varying exposures or potential “confounding by indication,” where patients with more severe comorbidities might not have received certain therapies.

Fourth, aside from treatment variables, our study did not consider all possible patient characteristics (such as specific biomarkers or socioeconomic factors). Future research should aim to include a broader range of parameters to further enhance the model’s predictive power.

Finally, a slightly better performance of the model was observed in the training set compared to the validation set, likely due to overfitting or differences in patient characteristics between the two sets. This aspect should be taken into account in practical applications.

### Future research directions

Future research should aim to validate this prognostic model in larger, multi-center scenarios. A deeper exploration of additional patient characteristics, biomarkers, and treatment strategies could refine the predictive model. Also, the integration of modern medical technologies, such as machine learning or artificial intelligence, could potentially enhance the model’s predictive accuracy.

### Conclusions

This study developed and internally validated a prognostic model designed to evaluate all-cause mortality risk at 6 months, 2 years, and 3 years after discharge in patients with HFmrEF following AMI. This model is predicated on six pivotal predictors significantly associated with mortality risk: age, prior stroke incidence, NYHA functional classification, hemoglobin levels, eGFR, and implementation of PPCI.

Our model demonstrated notable consistency and discrimination in the training and internal validation cohorts. While the model shows promise as a risk stratification tool, its generalizability remains to be confirmed. Rather than immediate clinical adoption, our findings primarily highlight the utility of these six predictors in the post-AMI HFmrEF population. External validation in large-scale, multi-center cohorts is mandatory to establish its robustness before it can be recommended for routine daily clinical practice.

## Data Availability

The datasets generated and/or analyzed during the current study are available from the corresponding author upon reasonable request.
